# Multiple magnetic scattering in small-angle neutron scattering of Nd–Fe–B nanocrystalline magnet

**DOI:** 10.1038/srep28167

**Published:** 2016-06-20

**Authors:** Tetsuro Ueno, Kotaro Saito, Masao Yano, Masaaki Ito, Tetsuya Shoji, Noritsugu Sakuma, Akira Kato, Akira Manabe, Ai Hashimoto, Elliot P. Gilbert, Uwe Keiderling, Kanta Ono

**Affiliations:** 1 National Institute for Materials Science, Tsukuba, 305-0047, Japan; 2 High Energy Accelerator Research Organization, Institute of Materials Structure Science, Tsukuba, 305-0801, Japan; 3 Elements Strategy Initiative Center for Magnetic Materials, Tsukuba, 305-0047, Japan; 4 Toyota Motor Corporation, Toyota, 471-8571, Japan; 5 Australian Nuclear Science and Technology Organisation, Bragg Institute, Lucas Heights, NSW 2232, Australia; 6 Helmholtz-Zentrum Berlin für Materialien und Energie, Berlin, 14109, Germany

## Abstract

We have investigated the influence of multiple scattering on the magnetic small-angle neutron scattering (SANS) from a Nd–Fe–B nanocrystalline magnet. We performed sample-thickness- and neutron-wavelength-dependent SANS measurements, and observed the scattering vector dependence of the multiple magnetic scattering. It is revealed that significant multiple scattering exists in the magnetic scattering rather than the nuclear scattering of Nd–Fe–B nanocrystalline magnet. It is considered that the mean free path of the neutrons for magnetic scattering is rather short in Nd–Fe–B magnets. We analysed the SANS data by the phenomenological magnetic correlation model considering the magnetic microstructures and obtained the microstructural parameters.

Small-angle neutron scattering (SANS) has been recognized as a powerful experimental technique to characterize permanent magnet materials^1^,^2^ such as Nd_2_Fe_14_B single crystal^3^, Nd-Fe-B sintered magnets^4^,^5^,^6^, hot-deformed nanocrystalline magnets^7^,^8^,^9^,^10^, and nanocomposites^11^,^12^. Because of the magnetic sensitivity and the high transparency of neutrons to matter, one can probe the magnetic properties of the materials to generate bulk averaged information. Moreover, the range of the scattering vector, *q*, matches the length scale of the microstructures and the magnetic domains in permanent magnet materials. These unique characteristics of SANS are complementary to experimental techniques such as transmission electron microscopy or X-ray microscopy that probe the local, rather than bulk, structure. For the development of high-performance permanent magnet materials, it is necessary to investigate magnetic structures and to reveal the origin of the coercivity. We have been reported so far SANS studies of Nd-Fe-B nanocrystalline magnets^7^,^8^,^9^,^10^. In previous papers, we qualitatively discussed the magnetic SANS in the magnetization reversal process. However, it is essential to extract the quantitative information such as particle-size distribution and magnetic correlation length during the magnetization reversal process from these SANS data.

Analysis of the small-angle X-ray or neutron scattering intensity considers, in general, the X-ray or neutrons to be singly-scattered, i.e. samples to be moderately thin. Such a single scattering approximation is invalid for thick samples in which the thickness exceeds the scattering mean free path (MFP)^13^. The scattering probability is proportional to the scattering cross-section of the material, and multiple scattering becomes noticeable for strongly scattering thick samples^13^,^14^. Multiple scattering smears the small-angle scattering patterns, and it blurs the “real”, single scattering, signals. For quantitative analysis of SANS data, the sample has to be thin enough to preserve the single-scattering approximation regime. However, on the other hand, the sample must be thick enough to be regarded as a bulk representative.

The multiple scattering in the small-angle approximation is usually described by the Molière’s theory which is based on the transport equation of particles^15^. The Molière’s theory is valid when the mean free path of the particles is large compared to the size of the samples. Schelten and Schmatz derived the analytical expression of multiple small-angle scattering by assuming the diffractive regime in which scattering by a single particle is kinematical^16^. More generalized dynamical treatment of multiple scattering is reported by Berk *et al*. including the refractive regime^17^,^18^. Mazumder and Sequeira intensively reported the multiple small-angle scattering effects for specific models^19^,^20^.

Multiple magnetic scattering with neutron beam transmission through ferromagnets was calculated^21^,^22^ and the interpretation of critical scattering in ferromagnets was also reported^23^. Šaroun formulated multiple small-angle neutron scattering including magnetic interaction based on the Molière’s theory^24^. From the viewpoint of SANS experiments, the multiple-scattering effect for nonmagnetic samples that only include nuclear scattering has been reported^25^,^26^,^27^. However, multiple magnetic scattering, which is important in magnetic SANS for magnetic materials, has only been reported particularly on beam broadening^28^,^29^,^30^,^31^. In permanent magnet materials such as Nd-Fe-B, multiple magnetic scattering should be raised by the large magnetic contrast of magnetic microstructures due to large spontaneous magnetization and high anisotropy.

In this study, we have investigated the influence of multiple scattering on the magnetic SANS from a Nd-Fe-B nanocrystalline magnet. Considering the Molière’s theory of multiple small-angle scattering, it is important to check the dependence of sample thickness on the mean free path of the neutrons. We clarify the multiple-scattering effect by either measuring the samples of different thicknesses using neutron beams with different wavelength^14^. SANS from a thermally demagnetized state of Nd-Fe-B nanocrystalline magnet were measured at room temperature (RT), which includes both nuclear and magnetic scattering arising from the magnetic domain walls, and also measured above the Curie temperature, *T*_C_, which only includes nuclear scattering. We reveal that significant multiple scattering exists in the magnetic scattering rather than the nuclear scattering. The phenomenological model fitting analysis was applied to the magnetic scattering intensities, and the microstructural parameters were obtained.

Before the results and discussion section, we introduce to the SANS intensity. The total elastic cross-section *d*Σ/*d*Ω for unpolarized neutron beam perpendicular to the external magnetic field (**k**_**0**_ ⊥ **H**_**0**_) is given as follows^2^:


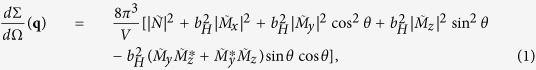


where *V* is the scattering volume, 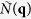 is the nuclear scattering amplitude, *b*_*H*_ is the atomic magnetic scattering amplitude per atomic magnetic moment (*b*_*H*_ = 2.91 × 10^8^ A^−1^m^−1^), 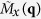, 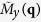, and 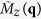 are the Fourier coefficients of the magnetization vector field, 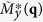 and 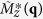 are complex conjugates of 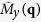 and 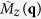, *θ* is the angle between **q** and **H**_**0**_. In the present experiment, the external magnetic field **H**_**0**_ = 0, however, the magnetization easy axis of the sample, i.e. nominal *c*-axis of the Nd_2_Fe_14_B grains is defined as a quantization axis. Therefore, *θ* measures the angle between **q** and the *c*-axis. In the thermally demagnetized state of the permanent magnet material, the SANS intensity both includes nuclear *d*Σ_nuc_/*d*Ω(**q**) and magnetic scattering cross-section *d*Σ_mag_/*d*Ω(**q**), thus, the observed scattering intensity *I*(**q**) is proportional to *d*Σ/*d*Ω(**q**). Above *T*_C_, the magnetization disappears and only the nuclear scattering term of Eq. 1 remains:


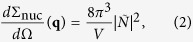


thus the nuclear scattering intensity *I*_nuc_(**q**) ∝ *d*Σ_nuc_/*d*Ω(**q**) is directly observed. On the other hand, the magnetic scattering cross-section *d*Σ_mag_/*d*Ω(**q**) is given as follows:


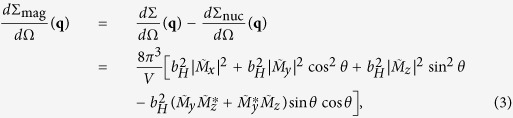


then the magnetic scattering intensity *I*_mag_(**q**) ∝ *d*Σ_mag_/*d*Ω(**q**) is obtained. Hereafter, the scattering vector **q** is represented as a projection **q** onto the detector plane, i.e. the *y*-*z* plane.

## Results and Discussion

### Experimental observation of magnetic multiple scattering in Nd-Fe-B nanocrystalline magnet

#### Nuclear and magnetic scattering

**Figure 1:**
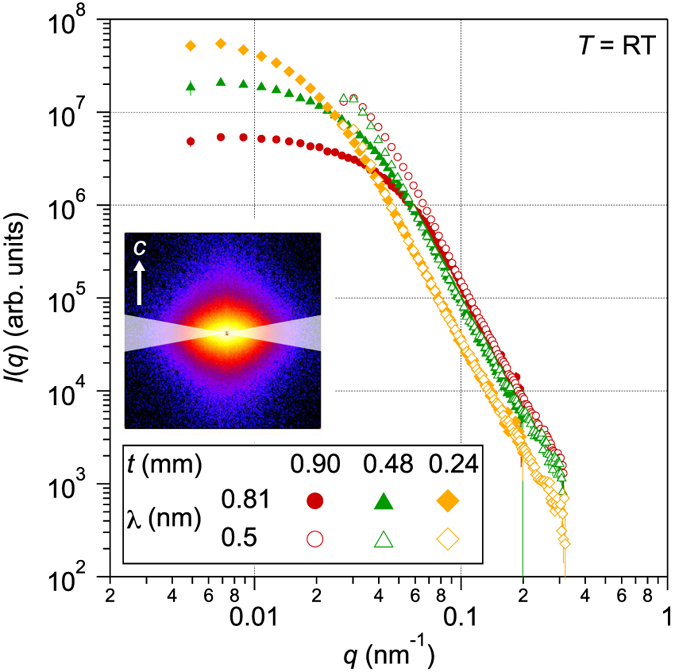
Scattering intensities for the Nd-Fe-B nanocrystalline magnet in the thermally demagnetized state. Scattering intensities *I*(*q*) for the Nd-Fe-B nanocrystalline magnet with different sample thickness *t* (*t* = 0.24, 0.48 and 0.90 mm) and neutron wavelength *λ* (*λ* = 0.5 and 0.81 nm) measured at room temperature. Sector averaging with a width of 20° was taken along the *c*-perpendicular directions of the SANS patterns. *I*(*q*) for *λ* = 0.5 nm (open symbols) are offset and overlapped to *I*(*q*) for *λ* = 0.81 nm (full symbols) for clarity. Typical SANS pattern for *t* = 0.90 mm and *λ* = 0.81 nm with 20°-width-sectors and *c*-axis is shown in the inset. The measurement was carried out on the QUOKKA instrument.

We performed sample-thickness- and neutron wavelength-dependent experiment, to reveal the extent to which multiple scattering in a Nd-Fe-B nanocrystalline magnet. Figure 1 shows scattering intensities *I*(*q*) for the Nd-Fe-B nanocrystalline magnet in the thermally demagnetized state with different sample thickness *t* (*t* = 0.24, 0.48 and 0.90 mm) and neutron wavelength *λ* (*λ* = 0.5 and 0.81 nm) measured at room temperature. For the thermally demagnetized state, *I*(*q*) includes both the nuclear scattering *I*_nuc_(*q*) and the magnetic scattering *I*_mag_(*q*) that arises from neutrons scattered at the magnetic microstructures. Typical SANS pattern for the thermally demagnetized state is shown in the inset of Fig. 1. SANS patterns for the Nd-Fe-B nanocrystalline magnet exhibit anisotropic intensity as a result of the anisotropic shape of Nd_2_Fe_14_B grains and the anisotropic contribution of the magnetic scattering along the *c*-perpendicular and *c*-parallel directions, respectively, as we reported previously^7^,^8^,^9^,^10^. The scattering intensity along the *c*-perpendicular direction (*θ* = 90°) includes the magnetic scattering from the magnetic microstructures along the nominal *c*-axis^9^. On the other hand, the scattering intensity along the *c*-parallel direction (*θ* = 0°) includes the magnetic scattering from the spin misalignment which comes from the orientational fluctuation of the Nd_2_Fe_14_B grains^10^. Hereafter, we focus on the scattering intensity along the *c*-perpendicular direction, which includes a large magnetic scattering contribution from the magnetic microstructures. Absolute intensities of *I*(*q*) for *λ* = 0.81 nm is comparable for different thicknesses because the intensities are normalized for *t*. By comparing *I*(*q*) for *λ* = 0.81 nm for different *t*, the intensity is suppressed for thicker samples in the lower *q* region. Inflection points of *I*(*q*) for *λ* = 0.81 nm and for *λ* = 0.5 nm shift toward higher *q* with increasing sample thickness. These are characteristics of multiple scattering^16^,^26^,^27^. For any thickness, *I*(*q*) for *λ* = 0.81 nm and *λ* = 0.5 nm overlap in the higher *q* (Porod) region.

#### Nuclear scattering

**Figure 2:**
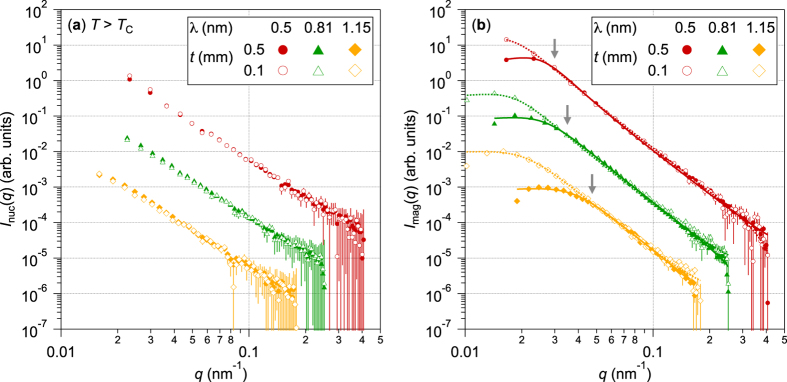
Nuclear and magnetic scattering intensities for the Nd-Fe-B nanocrystalline magnet. (**a**) Nuclear scattering intensities *I*_nuc_(*q*) at *T* > *T*_C_ and (**b**) magnetic scattering intensities *I*_mag_(*q*) for the Nd-Fe-B nanocrystalline magnet with different sample thickness *t* (*t* = 0.1 and 0.5 mm) and neutron wavelength *λ* (*λ* = 0.5, 0.81 and 1.15 nm). Sector averaging with a width of 20° was taken along the *c*-perpendicular directions of the SANS patterns. All *I*_nuc_(*q*) and *I*_mag_(*q*) are offset and *I*_nuc_(*q*) and *I*_mag_(*q*) for same *λ* are overlapped for clarity. In (**b**), arrows indicate the critical *q* values below which *I*_mag_(*q*) for *t* = 0.5 mm and *t* = 0.1 mm differ. Solid and dotted curves are the fitting results from the magnetic correlation model (Equation 6). The measurement was carried out on the V4 instrument.

The effect of multiple scattering on nuclear scattering was measured at elevated temperature. One can observe only nuclear scattering signals when measured above *T*_C_ because Nd_2_Fe_14_B becomes paramagnetic and magnetic interaction disappears. Figure 2(a) shows the nuclear scattering intensities *I*_nuc_(*q*) along the *c*-perpendicular direction for different *t* (*t* = 0.1 and 0.5 mm) and *λ* (*λ* = 0.5, 0.81 and 1.15 nm) observed at *T* > *T*_C_. Nuclear scattering intensities seem to be identical for all sample thicknesses and neutron wavelengths investigated. It indicates that the multiple-scattering effect on the nuclear scattering is negligible at least in the observed *q* region in this experiment (0.02–0.4 nm^−1^). It is suggested that the multiple-scattering behavior observed in the ferromagnetic state shown in Fig. 1 originates from the magnetic scattering.

#### Magnetic scattering

The magnetic scattering intensity *I*_mag_(*q*) was obtained by subtracting *I*_nuc_(*q*) from the scattering intensity obtained in the thermally demagnetized state *I*(*q*): *I*_mag_(*q*) = *I*(*q*) − *I*_nuc_(*q*). Figure 2(b) shows *I*_mag_(*q*) along the *c*-perpendicular direction for different *t* (*t* = 0.1 and 0.5 mm) and *λ* (*λ* = 0.5, 0.81 and 1.15 nm). Magnetic scattering intensities show different *q*-dependences for different sample thickness and neutron wavelength. Arrows in Fig. 2(b) indicate the critical *q* points below which *I*_mag_(*q*) for *t* = 0.5 mm and *t* = 0.1 mm behave differently. Suppression of the intensity in the low *q* region is marked for the thicker sample measured with longer wavelength neutrons which are one characteristic of the multiple-scattering effect. These characteristics are similar to multiple nuclear scattering in the literature^26^,^27^, however, the shape of *I*_mag_(*q*) for the thin sample (*t* = 0.1 mm) is largely independent of the *λ* investigated.

#### Multiple magnetic scattering

**Figure 3:**
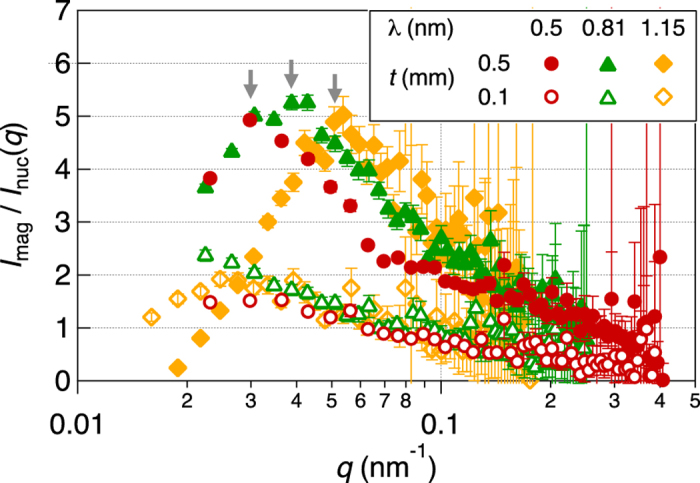
Magnetic to nuclear scattering intensity ratio for the Nd-Fe-B nanocrystalline magnet. Magnetic to nuclear scattering intensity ratio *I*_mag_/*I*_nuc_(*q*) are plotted for different sample thickness *t* (*t* = 0.1 and 0.5 mm) and neutron wavelength *λ* (*λ* = 0.5, 0.81 and 1.15 nm).

Figure 3 shows the magnetic to nuclear scattering intensity ratio *I*_mag_/*I*_nuc_(*q*) along the *c*-perpendicular direction for the Nd-Fe-B nanocrystalline magnet with different sample thickness *t* (*t* = 0.1 and 0.5 mm) and neutron wavelength *λ* (*λ* = 0.5, 0.81 and 1.15 nm). It is shown that the magnetic scattering intensities are 1–5 times larger than the nuclear scattering intensities especially in the low *q* region (below *q* ~ 0.1 nm^−1^). In particular, *I*_mag_/*I*_nuc_ for the thick sample (*t* = 0.5 mm) show maxima at specific *q* indicated by arrows in Fig. 3. These maxima coincide with the critical *q* points at which *I*_mag_(*q*) for *t* = 0.5 mm and *t* = 0.1 mm differ as shown in Fig. 2(b). The maximum *I*_mag_/*I*_nuc_ value of *I*_mag_/*I*_nuc_ ~ 5 does not depend on the neutron wavelength *λ*. These results suggest the most significant contribution to the multiple scattering effects in these ferromagnetic materials arises from the magnetic scattering associated with magnetic microstructures.

*I*_nuc_(*q*) and *I*_mag_(*q*) are proportional to the square of the nuclear scattering-length density (SLD) contrast (Δ*ρ*_nuc_)^2^ and that of the magnetic SLD contrast (Δ*ρ*_mag_)^2^, respectively. We estimate (Δ*ρ*_nuc_)^2^ and (Δ*ρ*_mag_)^2^ to discuss *I*_mag_/*I*_nuc_(*q*). It is known that the Nd-Fe-B nanocrystalline magnet is composed of Nd_2_Fe_14_B grains and grain boundary phases containing metallic Nd-rich phase^32^. We assume metallic hcp Nd for grain boundary phase. Nuclear SLD *ρ*_nuc_ for Nd_2_Fe_14_B (7.76 g/cm^3^) and hcp Nd (7.01 g/cm^3^) are evaluated to be 6.613 × 10^14 ^m^−2^ and 2.251 × 10^14 ^m^−2^, respectively. The square of the nuclear SLD contrast between Nd_2_Fe_14_B and hcp Nd is estimated to be (Δρ_nuc_)^2^ ≃ 1.90 × 10^29^ m^−4^. The square of the magnetic SLD is given as follows^2^:


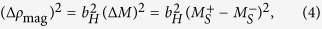


where 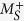 and 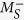 are the saturation magnetizations of the opposite magnetization directions. Magnetic SLD was calculated by assuming the saturation magnetization of Nd_2_Fe_14_B as *μ*_0_*M*_*S*_ = 1.61 T^33^. The contrast of the magnitude of the magnetization become *μ*_0_Δ*M* = 3.22 T at the magnetic domain walls, where *μ*_0_ is the magnetic permeability of the vacuum. For Nd_2_Fe_14_B, (Δ*ρ*_mag_)^2^ is estimated to be (Δρ_mag_)^2^ ≃ 5.56 × 10^29^ m^−4^ at the magnetic domain boundaries. The square of the magnetic SLD is about 3-times higher than that the nuclear SLD in Nd_2_Fe_14_B, however it does not explain why *I*_mag_(*q*) becomes up to 5-times higher than *I*_nuc_(*q*) as shown in Fig. 3. Therefore, it is suggested that the number of scattering events at the magnetic domain boundaries is much larger than that of the nuclear scattering at the grain boundaries.

By comparing *I*_nuc_(*q*) and *I*_mag_(*q*) for the same *q* range for the nuclear and magnetic scattering, it is evident that the multiple scattering more significantly affects the magnetic scattering in the Nd-Fe-B nanocrystalline magnet. The neutron MFP, i.e. the length on which the neutron beam intensity reduces to 1/*e*, in Nd_2_Fe_14_B (7.76 g/cm^3^) for the wavelength of 0.5, 0.81 and 1.15 nm, are 0.85, 0.54, and 0.39 mm, respectively, when only the nuclear scattering is considered. Thus, the sample thickness of *t* = 0.5 mm is thinner than or comparable to the MFP at *λ* = 0.5 nm and *λ* = 0.81 nm. However, significant magnetic multiple scattering were observed in these samples. Thus, the “magnetic” MFP, which appears to be shorter than “nuclear” MFP, should also be considered. To prevent the multiple magnetic scattering as well as multiple nuclear scattering, it is necessary to prepare sufficiently thin samples to preserve the single-scattering approximation regime demonstrated in this study. However, it is noted that preparing sufficiently thin samples results in its challenges regarding maintaining sample integrity, particularly if the material is brittle. Also, as the sample thickness is reduced, the ratio of surface-to-bulk increases and surface-dependent influences on the overall structure and the influence of both magnetic and nuclear scattering should be regarded.

Multiple magnetic scattering in SANS for the Nd-Fe-B nanocrystalline magnet is clearly experimentally observed. While we have discussed its origin within the context of the scattering length density and the neutron mean free path, more detailed theoretical studies for multiple magnetic scattering are desired.

### Analysis of SANS data with a phenomenological magnetic correlation model

It is essential to prepare thin enough samples that consider not only the nuclear MFP but also the magnetic MFP to suppress the multiple scatterings. However, this may be challenging to prepare sufficiently thin samples in the actual experiment for a variety of reasons. Also, there is a finite probability of multiple scattering for any sample with finite thickness because the scattering arises from a stochastic process. Therefore, it is important to explore an applicability of an analysis method of magnetic SANS in which this is considered. Silas and Kaler reported sample-thickness- and scattering-contrast-dependent, i.e. the multiple-scattering-dependent effects in SANS in microemulsions^34^. They plotted phenomenological parameters against the relative scattering probability and they obtained certain values on the extrapolating their data to zero thickness.

We propose a simple phenomenological magnetic correlation model for Nd-Fe-B nanocrystalline magnet. The phenomenological model is derived by considering features of the maze-like magnetic domains, i.e. alternating domains of opposite magnetization direction with periodicity and the shorter-range magnetic correlation within grains. The magnetic correlation function *γ*(*r*) is given as follows:


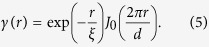


First term exp(−*r*/*ξ*) describes the magnetic correlation function from intra-grain magnetic interactions and the correlation length *ξ* correlates to the average radius of the Nd_2_Fe_14_B grains. The second term, the Bessel function *J*_0_(2*πr*/*d*), shows the magnetic domain structure originated from long-range magnetic interactions. Magnetic domains in the thermally demagnetized state of the permanent magnet materials exhibit maze-like or labyrinthine structures^35^,^36^,^37^,^38^ and the correlation function of the labyrinthine structure are known to be *J*_0_(2*πr*/*d*)^35^,^36^ where *d* is the periodicity. Equation (5) yields the scattering intensity as follows:


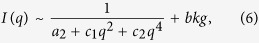


where *a*_2_, *c*_1_, *c*_2_, and bkg (incoherent background) are parameters. The parameters *d* and *ξ* are represented by


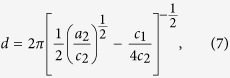


and


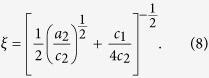


It should be noted that our magnetic correlation model for Nd-Fe-B nanocrystalline magnet is mathematically identical to the Teubner-Strey model which is usually adopted for microemulsion systems^39^. Application of the model to SANS of Nd-Fe-B nanocrystalline magnet has been reported previously^7^. We performed model fitting to the SANS data and the results of the model to *I*_mag_(*q*) are shown in Fig. 2(b) as solid and dotted curves.

**Figure 4:**
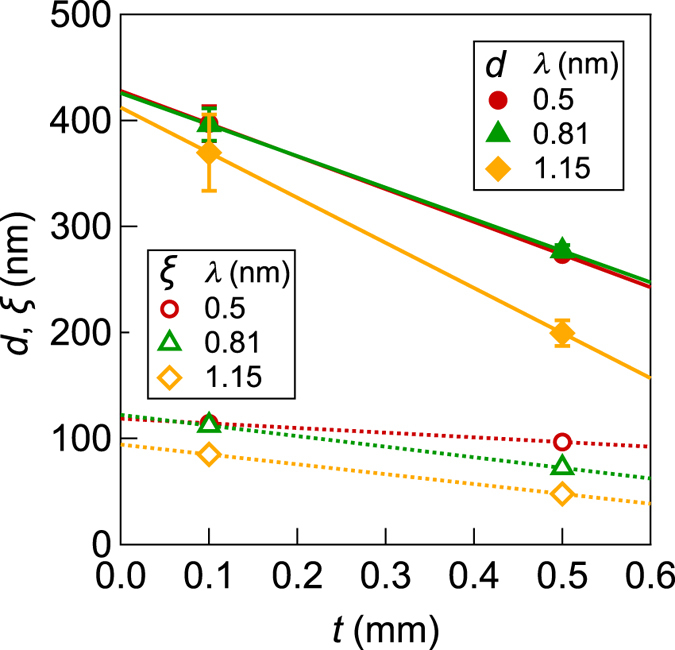
The magnetic periodicity and the correlation length versus the sample thickness. The magnetic periodicity *d* (full symbols) and the correlation length *ξ* (open symbols) are obtained from the fitting of the magnetic correlation model (Equation 6) to the magnetic scattering intensities *I*_mag_(*q*) for different sample thickness *t* and neutron wavelength *λ* shown in Fig. 2(b). Solid and dotted lines are linear regression of *d* and *ξ* for same *λ*.

The periodicity *d* and the correlation length *ξ* for different *λ* are plotted as a function of the sample thickness *t* in Fig. 4. For any neutron wavelength, *d* and *ξ* are smaller for the thicker sample than those for the thinner sample. Thus *d* and *ξ* are underestimated if one uses a SANS data with significant magnetic multiple scattering. Extrapolation of the linear regression of *d* and *ξ* to *t *→ 0 yields almost the same values for different *λ*. The extrapolation to *t* = 0 should, therefore, serve as reasonable approximations for the true values of *d* and *ξ* at the single-scattering regime^34^. The correlation length at *t* = 0, *ξ* ~ 110 nm, in the present case is interpreted as the radius of the Nd_2_Fe_14_B grains, and the diameter 2*ξ* ~ 220 nm is consistent with the diameter of Nd_2_Fe_14_B grains of 160–300 nm which is determined by transmission electron microscopy^32^. On the other hand, the magnetic periodicity at *t* = 0, *d* ~ 420 nm, is explained by magnetic domains formed by magnetically coupled grains, i.e. so-called interaction domains^38^,^40^. These results indicate the applicability of the phenomenological model and the extrapolation to the zero thickness to retrieve parameters for magnetic correlation function.

In conclusion, significant multiple-scattering effects have been observed in the magnetic scattering, rather than the nuclear scattering, in a Nd-Fe-B nanocrystalline magnet. A phenomenological model fitting approach was applied to the magnetic scattering and the magnetic periodicity, *d*, and the correlation length, *ξ*, were obtained. It is revealed that the analysis yields the anticipated values for the bulk magnetic domains in the thermally demagnetized state of Nd-Fe-B nanocrystalline magnet.

## Methods

### Sample preparations

Nd-Fe-B nanocrystalline magnet samples were made from rapidly quenched melt-spun ribbons. The melt-spun ribbons were crushed into powders of a few hundred micrometers and then sintered at 873 K under a pressure of 100 MPa. This sintered bulk was hot-deformed with a height reduction of ~80% to develop the (001) texture of the Nd_2_Fe_14_B phase. Nd_2_Fe_14_B grains are stacked along the *c*-directions with some degree of orientational fluctuation. Typical grain sizes are 160–300 nm and 50–110 nm in the *c*-perpendicular and *c*-parallel directions, respectively^32^. All samples were thinned to specific sample thickness, *t*, of between 0.1 and 0.9 mm. Samples were thermally demagnetized by heating up to 673 K (above *T*_C_ = 586 K of Nd_2_Fe_14_B^41^) in a vacuum furnace.

### SANS experiments

**Figure 5:**
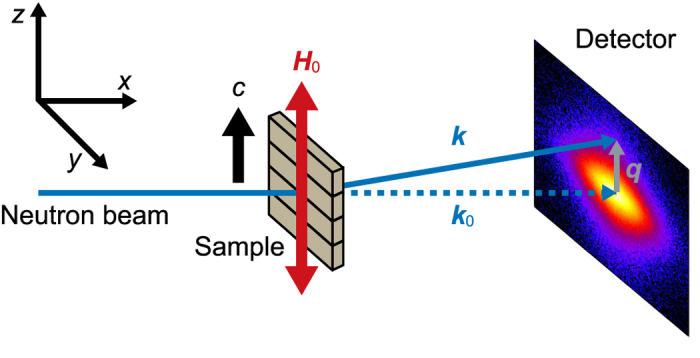
Schematic of the SANS experimental setup. Scattering vector **q** is defined as the difference between the wave vectors of the incident and transmitted neutron **k**_**0**_ and scattered neutron **k**. External magnetic field direction **H**_**0**_ and the nominal *c*-axis are parallel, both of those are perpendicular to **k**_**0**_.

Small-angle neutron scattering experiments were performed on the QUOKKA instrument at the OPAL research reactor at the Australian Nuclear Science and Technology Organisation (ANSTO)^42^ and on the V4 instrument at the BER-II research reactor at Helmholtz-Zentrum Berlin (HZB)^43^. Figure 5 shows the schematic of the SANS experimental setup. Unpolarized neutron beams with wavelength, *λ*, of 0.5, 0.81 and 1.15 nm were used. The sample temperature *T* was set to RT and above *T*_C_ of Nd_2_Fe_14_B. Data reduction was performed using the NCNR SANS reduction procedure for IGOR Pro^44^ and BerSANS^45^, respectively.

## Additional Information

**How to cite this article**: Ueno, T. *et al*. Multiple magnetic scattering in small-angle neutron scattering of Nd–Fe–B nanocrystalline magnet. *Sci. Rep.* **6**, 28167; doi: 10.1038/srep28167 (2016).

## References

[b1] Michels A. & Weissmüller J. Magnetic-field-dependent small-angle neutron scattering on random anisotropy ferromagnets. Rep. Prog. Phys. 71, 066501 (2008).

[b2] Michels A. Magnetic small-angle neutron scattering of bulk ferromagnets. J. Phys. Condens. Matter 26, 383201 (2014). 25180625 10.1088/0953-8984/26/38/383201

[b3] Kreyssig A. . Probing Fractal Magnetic Domains on Multiple Length Scales in Nd_2_Fe_14_B. Phys. Rev. Lett. 102, 047204 (2009). 19257472 10.1103/PhysRevLett.102.047204

[b4] Périgo É. A., Gilbert E. P., Metlov K. L. & Michels A. Experimental observation of magnetic poles inside bulk magnets via q ≠ 0 Fourier modes of magnetostatic field. New J. Phys. 16, 123031 (2014).

[b5] Périgo E. A., Gilbert E. P. & Michels A. Magnetic SANS study of a sintered Nd-Fe-B magnet: Estimation of defect size. Acta Mater. 87, 142–149 (2015).

[b6] Périgo E. A. . Magnetic microstructure of a textured Nd-Fe-B sintered magnet characterized by small-angle neutron scattering. J. Alloys Compd. 661, 110–114 (2016).

[b7] Yano M. . Magnetic Reversal Observation in Nano-Crystalline Nd-Fe-B Magnet by SANS. IEEE Trans. Magn. 48, 2804–2807 (2012).

[b8] Yano M. . Investigation of coercivity mechanism in hot deformed Nd-Fe-B permanent magnets by small-angle neutron scattering. J. Appl. Phys. 115, 17A730 (2014).

[b9] Ueno T. . Magnetization Reversal Process in Pr-Cu Infiltrated Nd-Fe-B Nanocrystalline Magnet Investigated by Small-Angle Neutron Scattering. IEEE Trans. Magn. 50, 2103104 (2014).

[b10] Saito K. . Magnetization reversal of a Nd-Cu-infiltrated Nd-Fe-B nanocrystalline magnet observed with small-angle neutron scattering. J. Appl. Phys. 117, 17B302 (2015).

[b11] Bick J.-P. . Magnetization reversal in Nd-Fe-B based nanocomposites as seen by magnetic small-angle neutron scattering. Appl. Phys. Lett. 102, 022415 (2013).

[b12] Bick J.-P. . Exchange-stiffness constant of a Nd-Fe-B based nanocomposite determined by magnetic neutron scattering. Appl. Phys. Lett. 103, 122402 (2013).

[b13] Mazumder S. & Sequeira A. Multiple small-angle scattering–A review. Pramana-J. Phys. 38, 95–159 (1992).

[b14] Pauw B. R. Everything SAXS: small-angle scattering pattern collection and correction. J. Phys. Condens. Matter 25, 383201 (2013). 23988669 10.1088/0953-8984/25/38/383201

[b15] Bethe H. A. Molière’s Theory of Multiple Scattering. Phys. Rev. 89, 1256–1266 (1953).

[b16] Schelten J. & Schmatz W. Multiple-Scattering Treatment for Small-Angle Scattering Problems. J. Appl. Cryst. 13, 385–390 (1980).

[b17] Berk N. F. & Hardman-Rhyne K. A. Analysis of SAS Data Dominated by Multiple Scattering. J. Appl. Cryst. 21, 645–651 (1988).

[b18] Allen A. J. & Berk N. F. Analysis of Small-Angle Scattering Data Dominated by Multiple Scattering for Systems Containing Eccentrically Shaped Particles or Pores. J. Appl. Cryst. 27, 878–891 (1994).

[b19] Mazumder S. & Sequeira A. Multiple small-angle scattering from a polydispersed random medium. Phys. Rev. B 39, 6370–6373 (1989). 10.1103/physrevb.39.6370 9947272

[b20] Mazumder S. & Sequeira A. Multiple small-angle scattering from a bidisperse Markov medium. Phys. Rev. B 41, 6272–6277 (1990). 10.1103/physrevb.41.6272 9992872

[b21] Halpern O. & Holstein T. On the Passage of Neutrons Through Ferromagnets. Phys. Rev. 59, 960–981 (1941).

[b22] Hiismaki P. A SANS filter as a white neutron beam polariser. J. Phys. D: Appl. Phys. 16, 2405–2413 (1983).

[b23] Toperverg B. P., Runov V. V., Gukasov A. G. & Okorokov A. I. The investigation of double critical magnetic scattering in polarized neutron experiments. Phys. Lett. A 71, 289–291 (1979).

[b24] Šaroun J. Evaluation of multiple small-angle neutron scattering including magnetic interactions. J. Appl. Cryst. 40, s701–s705 (2007).

[b25] Long G., Krueger S. & Allen A. Multiple Small-Angle Neutron Scattering. J. Neutron Res. 7, 195–210 (1999).

[b26] Radliński A. P. . Fractal Geometry of Rocks. Phys. Rev. Lett. 82, 3078–3081 (1999).

[b27] Radlinski A. P. Small-Angle Neutron Scattering and the Microstructure of Rocks. Rev. Mineral. Geochem. 63, 363–397 (2006).

[b28] Bogdanov S. G., Valiev E. Z. & Menshikov A. Z. On the nature of giant small-angle neutron scattering in Fe_3_Pt. Solid State Commun. 76, 809–813 (1990).

[b29] Menshikov A. Z., Bogdanov S. G. & Skryabin Y. N. Multiple small-angle neutron scattering in ferromagnets. Physica B 234–236, 584–585 (1997).

[b30] Bogdanov S. G. & Menshikov A. Z. Multiple small angle neutron scattering: Numerical analysis and experiment. Physica B 276–278, 79–80 (2000).

[b31] Woo W., Em V., Shin E., Mikula P. & Ryukhtin V. Influence of multiple small-angle neutron scattering on diffraction peak broadening in ferritic steel. J. Appl. Cryst. 48, 350–356 (2015).

[b32] Sepehri-Amin H. . High-coercivity ultrafine-grained anisotropic Nd-Fe-B magnets processed by hot deformation and the Nd-Cu grain boundary diffusion process. Acta Mater. 61, 6622–6634 (2013).

[b33] Sagawa M., Fujimura S., Yamamoto H., Matsuura Y. & Hirosawa S. Magnetic properties of rare-earth-iron-boron permanent magnet materials. J. Appl. Phys. 57, 4094–4096 (1985).

[b34] Silas J. A. & Kaler E. W. Effect of multiple scattering on SANS spectra from bicontinuous microemulsions. J. Colloid Interface Sci. 257, 291–298 (2003). 16256483 10.1016/s0021-9797(02)00059-0

[b35] Seul M. & Andelman D. Domain Shapes and Patterns: The Phenomenology of Modulated Phases. Science 267, 476–483 (1995). 17788780 10.1126/science.267.5197.476

[b36] Le Berre M. . Example of a chaotic crystal: The labyrinth. Phys. Rev. E 66, 026203 (2002). 10.1103/PhysRevE.66.026203 12241263

[b37] Ono K. . Element-Specific Magnetic Domain Imaging of (Nd, Dy)-Fe-B Sintered Magnets Using Scanning Transmission X-Ray Microscopy. IEEE Trans. Magn. 47, 2672–2675 (2011).

[b38] Thielsch J. . *In situ* magnetic force microscope studies of magnetization reversal of interaction domains in hot deformed Nd-Fe-B magnets. J. Appl. Phys. 111, 103901 (2012).

[b39] Teubner M. & Strey R. Origin of the scattering peak in microemulsions. J. Chem. Phys. 87, 3195–3200 (1987).

[b40] Khlopkov K. . Evolution of interaction domains in textured fine-grained Nd_2_Fe_14_B magnets. J. Appl. Phys. 102, 023912 (2007).

[b41] Hirosawa S. . Magnetization and magnetic anisotropy of R_2_Fe_14_B measured on single crystals. J. Appl. Phys. 59, 873–879 (1986).

[b42] Gilbert E. P., Schulz J. C. & Noakes T. J. ‘Quokka’–the small-angle neutron scattering instrument at OPAL. Physica B 385–386, 1180–1182 (2006).

[b43] Keiderling U. & Wiedenmann A. New SANS instrument at the BER II reactor in Berlin, Germany. Physica B 213–214, 895–897 (1995).

[b44] Kline S. R. Reduction and analysis of SANS and USANS data using IGOR Pro. J. Appl. Cryst. 39, 895–900 (2006).

[b45] Keiderling U. The new ‘BerSANS-PC’ software for reduction and treatment of small angle neutron scattering data. Appl. Phys. A 74, S1455–S1457 (2002).

